# Periscope of Dermatology

**Published:** 1898-05-28

**Authors:** 


### PERISCOPE OF DERMATOLOGY.

**Leprosy.**—The following are the chief conclusions of the recent International Congress on Leprosy at Berlin[1]: The bacillus lepræ, discovered by Hansen, is the cause of the disease. The conditions of its existence and development, and the mode of its entry into the body, are still unknown, but it seems that opinion may soon be unanimous. The disease occurs only in man. The contagious nature of the disease was recognised by all, and it was agreed that every leper is dangerous to his neighbours, and the danger increases in proportion to the intimacy of his relations with them, and the neglect of sanitary precautions. Although this refers chiefly to the poorer classes, it must be remembered that leprosy has been transmitted to the well-to-do. The view that leprosy is hereditary continues to lose ground. The only good effects of treatment are palliative, and even the serum treatment had yielded negative results. In view of its incurability, of the suffering which it involves, and of its contagious nature, it follows that isolation is the only radical and effective means of suppressing the disease. In Norway a law exists which compels the isolation, even againt their will, of patients who are poor enough to make voluntary isolation impossible. The Congress unanimously accepted the following recommendations: (1) In all countries in which leprosy appears in certain localities or more widely distributed, isolation is the best means of preventing its spread. (2) The system of compulsory notification, of supervision, and of isolation as carried out in Norway, is to be recommended to all nations possessing the means of giving force to it. (3) It must be left to the law, after consultation with the sanitary authorities, to determine the necessary regulations, which must be adapted to the social status of the sufferer.

Impey,[2] who was medical superintendent at the Robben Island Asylum, maintains that anæsthetic leprosy is not contagious since no nodules or tubercles are found in these cases, and the bacilli are only found in the nerves. He agrees with those authorities who consider that in anæsthetic leprosy all the tissues except the nerves are immune against the bacilli; in consequence of this immunity the bacilli cannot escape from the nerves and so cannot infect other parts of the body. Those cases of anæsthetic leprosy in which fresh outbreaks of the disease occur are, in his opinion, due to reinfection of an open sore by a fresh dose of leprosy bacillus. He therefore considers that it is not only unnecessary, but absolutely wrong, to segregate anæsthetic lepers with those suffering from the tubercle variety, and he urges that sufferers from anæsthetic leprosy may be left at large without any danger to the rest of the community.

Thomspon,[3] in a paper on the history and prevalence of leprosy in Australia, does not think that the disease was ever indigenous in any part of the country. As the result of his investigations he states the following facts: (1) Although lepers were imported to Victoria during a long period of years, during which time their movements were entirely unrestricted, no Victorian native has ever been attacked; moreover, the disease died out when restrictive measures were taken against the liberty of lepers; (2) although many lepers had been imported into all parts, and cases of the disease had occurred among whites living north of the 35th parallel of south latitude, no native white had ever been attacked who lived south of this latitude.

Jeanselme and Laurens[4] found that there was some affection of the nose or upper air passages in three-fifths of the cases of leprosy examined. A persistent cold in the head, obstruction of the nostrils by crusts, and some epistaxis often constitutes the first manifestation of leprosy. It is, therefore, suggested that the bacillus gains entrance into the system through a slight abrasion of the pituitary membrane. Accompanying the coryza and epistaxis, there is marked anæsthesia of the air passages. Bacilli were nearly always found in the nasal discharge.

Sticker[5] relates the experience of leprosy which he had gained in Egypt and India. He points out that the disease usually attacks the nasal mucous membrane first, and the part most frequently attacked is the septum. The disease spreads from the nose to other parts by the lymphatics and more rarely by the blood. In only four out of 143 cases were any bacilli found in the blood. He urges the importance of local treatment not only on account of the patient himself but also to prevent the spread of the disease.

Von Delupes[6] describes two cases of leprosy observed in Lissa, both of which were in females. In the first case, a girl of eighteen, the disease had been communicated by a woman, aged 26, who lived in the next house, the partition between the houses consisting of boards only, in a bad state of repair. The disease commenced with an eruption on the forearms. Strict isolation and disinfection of the dwelling was recommended.

Meschede[7] states that he had observed acute hallucinary monomania in one of the 12 lepers in the Memel district. The hallucinations were tactile, the patient complaining of being beaten, cut, and burnt; they disappeared in three and a half months.

Schönfeld[8] states that senile dementia is common in lepers, but he has never observed mental disease which could be traced to leprosy.

Besnier,[9] in a paper on the etiological characters of leprosy, does not consider that heredity plays much part in the transmission of the disease, but holds that it must always be communicated by contact. He does not believe in the reported long periods of incubation. He also points out that the healthy children of leprous parents may show complete immunity to the disease.

Long and Valençay[10] report a case of leprosy which occured in a native of Brittany, who had never left his country. He presented typical signs of leprosy of a tuberculo-anæsthetic type, and the diagnosis was completed by the discovery of quantities of bacilli in one of the nodules.

Cantlie[11] considers leprosy as essentially a Chinese disease extending from its focus in the south-east provinces of China, to every region visited by the lower class of Chinaman. In countries into which it has been imported by the Chinese, their expulsion is followed by the disappearance of the disease, since the aborigines usually escape. Malays and the Japanese are not so liable to the disease as are the Chinese.

Heath[12] also contributes an exhaustive paper on the origin and the spread of leprosy.

**Eczema.**—Shoemaker[13] describes a case of rhagades of the hand due to chronic eczema. He points out that the fissures in the skin are due to muscular action. As a rule, they affect the epidermis alone, but they may extend into the corium, when they frequently give rise to bleeding. They occur whenever the elasticity of the skin is impaired by inflammatory infiltration. He recommends the application of the following ointment: ℞ Acid salicyl. ʒ½, Bismuthi subnitratis. ʒi, creosoti ♏5, ung. zinci ox. benzoat., ung. aquæ rosæ āā℥½, M. fiat. ung.

Jacquet[14] employs linear scarification in the treatment of chronic eczema. The surface is thoroughly cleansed by repeated applications of potatoe poultices, and then scarified with a sharp point in parallel lines one to two mm. apart. Six or seven sittings, according to the nature of the case, are required, the lichenoid forms being most benefited by the treatment. In 11 cases, all of which had proved refractory to other treatment, a cure resulted.

Aubert[15] recommends the application of picric acid in acute eczema accompanied by swelling, superficial ulceration, and weeping. It immediately stops the itching and the inflammation subsides, a scab forms by coagulation of the discharge, under which healing quickly takes place. It has no effect on chronic eczema. It is applied as follows: A solution of 12 grammes of picric acid in one litre of tepid boiled water is painted on the affected area and the part wrapped in lint soaked in the same solution; no oilsilk, however, should be used. The dressing is removed every three days and a cure is effected in from 10 to 15 days.

Galgey[16] relates the case of a man, aged 42, residing at St. Lucia, who developed elephantiasis of the left leg in consequence of eczema of many years' standing. Filiaria were found in the blood. The eczema was cured by the application of an ointment composed of ichthyol ʒi, acid salicyl. ʒi, lanolin and ol. olivæ āāʒ10. The elephantiasis, however, was unaffected.

Kistler[17] treats infantile eczema, internally, by one or two grain doses of calomel given twice a week, and externally he applies either the benzoated oxide of zinc ointment or the following: ℞ Acid salicyl, two parts; bismuthi subnitratis, 40; corn starch, 15; ointment of rose water, 100 parts; M. fiat ung. These salves are repeatedly applied until a thick white coating forms on the surface. Frequent washing with soap and water is to be strenuously interdicted, and arsenic in one to three minim doses of Fowler's solution is a useful adjunct to the treatment.

1 Glasg. Med. Journ., Mar., 1898, p. 236. 2 Lancet, Oct. 30, 1897, p 1,127; ditto, Sept. 25, 1897, p. 789. 3 Ditto, Mar. 5, 1898, p. 627. 4 Med. Week, July 30, 1897. 5 Epit. Brit. Med. Jour., Dec 25, 1897, No. 526. 6 Brit. Jour. of Derm., Jan. 1898, p. 33. 7 Ditto, ditto, p. 34. 8 Ditto, ditto. 9 Ditto, ditto. 10 Practitioner, Nov., 1897, p. 531. 11 Lancet, Jan. 1, 1898, p. 32. 12 Birm. Med. Review, Aug. 1897, p. 76. 13 Med. Bull., Dec. 1897, p. 447. 14 Epit. Brit. Med. Jour., Mar. 12, 1898, No. 226. 15 Ditto, Feb. 5, 1898, No. 179. 16 Ditto, Dec. 4, 1897, p. 1,641. 17 Med. Rec., Feb. 12., 1898, p. 231.

